# Supplementary material for: Identification of photoperiod-induced specific miRNAs in the adrenal glands of Sunite sheep (Ovis aries)
Source: Front Vet Sci. 2022 Jul 22;9:888207. doi: 10.3389/fvets.2022.888207 (PMC9354845; doi:10.3389/fvets.2022.888207)
Supplement: Supplementary file 1 [file Data_Sheet_1.ZIP › Supplementary file/miRDeep2 file.zip!/miRDeep2 file/Novel_Structure/SPLP42A1/Novel_364.pdf]

Provisional ID	:	NC_019459.2_3965
Score total	:	2.4
Score for star read(s)	:	-1.3
Score for read counts	:	0
Score for mfe	:	2.1
Score for randfold	:	1.6
Score for cons. seed	:	
Total read count	:	17
Mature read count	:	12
Loop read count	:	0
Star read count	:	5

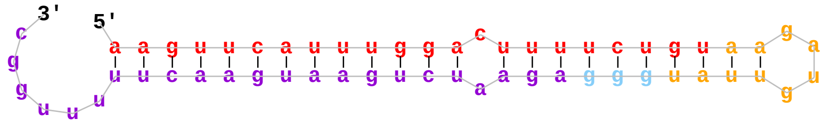

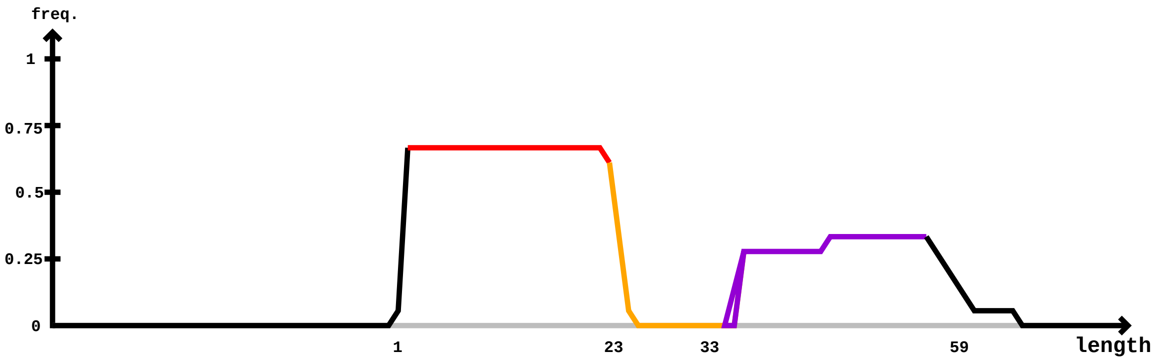

Mature **Star**

```
5'- aguuuguuuauucuauauauauuggauuggucaaaaaguucauuuggacuuuucuguaagauguuaugggagaaucugaaugaacuuuuuggccaaucccaauaagcagu   -3'    obs
    aguuuguuuauucuauauauauuggauuggucaaaaaguucauuuggacuuuucuguaagauguuaugggagaaucugaaugaacuuuuuggccaaucccaauaagcagu          exp
    ...(((((((((...........(((((((((((((((((((((((((.((((((((((....)))))))))).))))))))))))))))))))))))).))))))))).   reads   mm   sample
    ..................................aaaguucauuuggGcuuuucugu.....................................................   1       1    seq
    ...................................aaguucauuuggGcuuuucug......................................................   1       1    seq
    ...................................aaguucauuuggGcuuuucugu.....................................................   9       1    seq
    ...................................aaguucauuuggGcuuuucuguaa...................................................   1       1    seq
    ......................................................................agaaucugaaugaacuuuuu....................   1       0    seq
    ......................................................................agaaucugaaugaacuuuuugg..................   2       0    seq
    ......................................................................aAaaucugaaugaacuuuuuggc.................   2       1    seq
    ...............................................................................augaacuuuuGggccaaucc...........   1       1    seq
```
